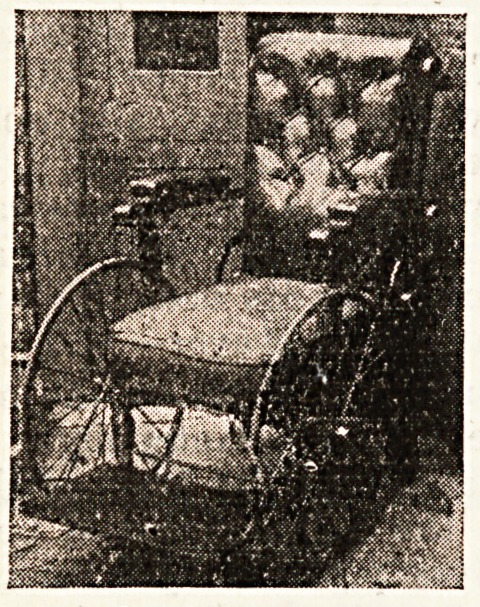# The Institutional Worker

**Published:** 1913-09-13

**Authors:** 


### INSTITUTIONAL WORKER

Being a Special Supplement to "The Hospital"

### OUR BUREAU OF INFORMATION.

### Rules for Correspondents.

1. Every letter must be accompanied by the coupon to be cut from the back cover (inside page) of The Hospital, current issue, and must contain the name and address of the correspondent with pseudonym for publication if desired. Replies by post cannot be given, save under exceptional circumstances at the Editor's discretion.

2. Letters from Approved Homes in reply to special needs published in the Bureau should state terms and full particulars, and be sent prepaid under cover to the Editor of the Bureau with name written across coupon for identification.

3. Proprietors of Homes which have not yet been entered on the List of Approved Homes, but have spare accommodation likely to suit special needs, are invited to write for an application form for registration. The fee for registration, which includes two announcements of the Home in the Bureau and other privileges, is 10s.

4. All communications to be addressed to the Editor of The Hospital, 28 Southampton Street, Strand, London, W.C., and marked "Bureau of Information."

### INSTITUTIONAL FACTS AND FIGURES.

### Forced Draught Apparatus and Boiler Efficiency.

There are many factors which go towards the attainment of boiler efficiency, and these are of necessity most familiar to the engineer. In view, however, of the great expense of coal and the costliness and importance of boilers the hospital secretary also should have some knowledge of these matters. Foremost amongst them is the question of furnace draught. An ordinary boiler furnace with "natural draught" has a bridge or baffle plate fixed at the end of the grate, and an open ash-pit supplies the air necessary for combustion. The air ascends through the apertures of the grate to the furnace, combining chemically with the gases emanating from the coal, and the combined product passes over the bridge into the combustion chamber, whence it travels through the flues to the base of the chimney. Its velocity is here arrested and regulated by means of a suitable damper. One means of increasing the draught is, of course, to increase the height of the chimney, but a tall chimney stack is not a usual or a desirable adjunct to a hospital. In this connection it may be remarked that when the new buildings of King's College Hospital are viewed from the back, in Ruskin Park, a prominent feature is a chimney stack about fifty feet high rising from the engine-house. This, however, is not solely for purposes of draught, for Professor Capper has had a forced draught apparatus fitted to the new furnaces.

#### Types of Apparatus.

A cheap and simple way to increase the rate of combustion is to fix a steam-jet in the ash-pit and to reduce the grate area to the area of the furnace front, the object of the latter being to diminish the air space. The usual apparatus for forced draught, however, is a large and costly affair, ranging in price according to the make and size of the boiler from £50 to £400. When such an apparatus is fitted the ash-pit is enclosed to prevent the egress of air, and a fan, preferably driven by an independent motor, is used to force the air (previously heated to about 250° by passing it over the flues) into the furnace. Suitable valves are fitted to the furnace fronts to regulate the air pressure either at the top or bottom of the furnace and to prevent an outrush of flame upon the doors being opened for stoking.

#### A Saving of Fuel.

The advantage of forced draught is, of course, that a higher rate of combustion is obtained, and the steam-raising power of the fuel thereby increased. Some makers claim for their apparatus that it increases the evaporative capacity of each boiler from 40 to 50 per cent. It is easy to see that if this claim is at all well founded a very great saving of fuel takes place. This saving can be realised either by decreasing the consumption of coal or by using a much cheaper fuel. The second course is the one usually adopted, and the fuel used is small coal or coal-dust.

#### Objections to Small Fuel.

In this connection the actual experience of a London hospital is instructive. The hospital is one of 150 beds, and the apparatus cost £75. While it was in use the saving in fuel was reckoned at 30s. to 35s. per week. Coal-dust or slack was used, and after a time complaints of smuts and dirt in the atmosphere began to be general throughout the hospital. These ultimately became so persistent that the apparatus was "scrapped." Whether such a drastic course was necessary may be doubted, for in view of the high temperature at which air is fed into the furnace with a forced draught apparatus, and of the constancy of pressure and facilities for regulating the amount of air in relation to the gases, perfect combustion should be obtained. In other words, the full thermal value of the fuel should accrue, so that the fuel having the highest thermal value would be the most desirable. A cheap coal, or coal-dust, contains a large percentage of ash and incombustible matter, all of which must be regarded as wasted from the steam-raising point of view. Moreover, in burning inferior coal difficulty is apt to arise from the fuel matting together and clinkering on the bars, thus retarding the free circulation of air. On the point simply of fuel consumption there can be no doubt that forced draught leads to economy in this direction, but whether this economy is sufficient to justify the prime cost will depend upon the circumstances of each case.

#### Importance of Efficient Stoking.

Whether forced draught be in use or not it is important to remember that the efficiency of the boiler depends to a great extent upon the stoker. The fire should be evenly laid and maintained at an average depth on the bars of about four inches. Constant attention is necessary to prevent this bed from burning into holes, while the fire bars must be kept clean and the damper regulated to give a maximum of air just after firing. If too little air be admitted imperfect combustion takes place and a black smoke emanates from the chimney; this is extremely undesirable, for not only is a wastage of fuel indicated, but the smoke may prove a nuisance to the hospital as well as to the neighbourhood. There is an apparatus on the market which automatically opens and shuts the damper as necessary, and the makers undertake to demonstrate free of cost a saving in fuel of at least 7 per cent. from its use. The price of the apparatus, however—£55—is high.

The supply of water to the boiler is, of course, an important point. It must be kept at a constant level, and before it enters the boiler it should be heated as much as possible by means of any available waste heat or steam. The water itself should not be hard, as otherwise the salts of lime will be precipitated on the inner surfaces of the boiler, causing a crust which will be very costly to remove, and more costly if allowed to remain.

Finally, the opinion of the engineer should always be taken before a steam coal is decided on, as the efficiency of the boiler will depend very largely upon the quality of the fuel. The truth of this has recently been brought to notice at the inquiry concerning the railway disaster at Aisgill. Insufficiency of steam in a hospital may lead to many complaints and much unpleasantness, while the boiler, the engineer, and the stoker may be severally and jointly blamed for what is in reality due to false economy in the matter of coal.

### Questions for September.

**No. 1 Annual Cleaning.**

**What must be the essential details involved in the annual cleaning and painting, and what is the best method of supervising the work? [Detailed information as to the type and relative cost of paints and enamels found by experience to be most suitable should be given, and any suggestion on the best way to make the supervision effective will be favourably regarded in selecting the replies.]**

**No 2. X-Ray Apparatus**

**Give particulars of the X-ray apparatus in use at your institution, the names of makers and cost of installation. State the number of each class of cases dealt with, and give a detailed account of the annual cost of maintenance.**

RULES.

The following rules must be observed:—

1. Contributions must be written on one side of the paper. They must bear the name and address of the sender and be accompanied by coupon to be cut from the back cover (inside page) of the current issue of The Hospital. A pseudonym must be chosen if the name is not to be published.

2. They must be addressed to the Editor of The Hospital, 28 & 29 Southampton Street, Strand, London, W.C., so as to reach him before the end of the month, and must be marked in the left-hand corner "Facts and Figures."

**A minimum payment of Half a Guinea will be made for each published answer.**

### A New Invalid Chair Offered.

The owner of an invalid chair made by Foot and Son, of New Bond Street, is anxious to sell it. It cost £12 14s. 6d. and has never been used because the owner is too crippled with rheumatic arthritis to leave her bed. She is in poor circumstances, and would be glad to sell the chair, which was a gift, at a considerable reduction. The accompanying sketch shows the chair in an upright position. The back can be adjusted to any angle, and there is a wheel at the rear to enable the chair to be pushed or rolled. Offers should be addressed to B. M., care of the Editor, and the words "Invalid Chair" written in the left-hand corner of the envelope.

### Approved Home.

We have pleasure in adding the following Home to our Approved List:—

**Surrey.**—Penshurst. Mount Pleasant, Weybridge. Miss Lydia Sheldrake receives into her Private Home a few blind, backward, or mentally defective children. Home life, with individual training and teaching. Terms moderate and inclusive.

### THE EDITOR'S LETTER-BOX.

*THE SICK AND IN NEED.*

### Home Required for Payment of Services.

A middle-aged lady who, as a result of a series of illnesses, is now suffering from loss of strength, seeks a nursing home willing to receive her in return for slight services and a weekly payment of 10s. She is not an invalid in the generally accepted sense, but she is unequal to much exertion or prolonged effort. Though not a trained nurse, she has had slight nursing experience, and prior to her breakdown held, amongst other positions, that of lady-superintendent to a small private home. Address replies to "Cia," 173.

### Nervous Breakdown.

We suggest that you apply to the following institutions on behalf of your step-sister, who is suffering from nervous breakdown and requires a change and fresh air:—Frederica Countess of Scarbrough's Home, Skegness, Lincs., 7s. 6d. a week; In Memoriam Cottage Home, Wentbridge, near Pontefract, from 5s. a week; and Wakefield Convalescent Home and Guild of Pity, Lupset, near Wakefield, free with subscriber's letter.—"Helpful."

### Bath-chair Required.

We should be glad to hear from any of our readers able to offer a bath-chair to a poor woman who is a great sufferer from arthritic rheumatism; a friend would gladly pay carriage. Address replies to J.W., 174.

*EMPLOYMENT AND TRAINING.*

### Male Nurse.

The choice between the training schools you refer to depends upon what branch of nursing the man desires to take up. If he wishes to obtain the Medico-Psychological certificate and become a fully trained nurse, he must serve for three years in a mental hospital (or asylum). The training at the National Hospital for Paralysed and Epileptic consists of instruction in practical nursing, massage, and electrical treatment. Candidates, if accepted as probationers, are required to serve the hospital for two years, and at the conclusion of this period are granted a certificate upon examination. Those who hold this certificate can join the private nursing staff. The fees of well trained mental nurses and masseurs vary from two to three guineas a week.—"Scot."

### Naval Nursing Service.

A candidate for Queen Alexandra's Royal Naval Nursing Service must be between 25 and 30 years of age, and produce a certificate of training for at least three years at a large general hospital in the United Kingdom. A form of application may be obtained from the Director-General, Medical Department of the Navy, 18 Victoria Street, S.W. If you will study the advertisement columns of *The Nursing Mirror* you will note that vacancies for staff nurses and sisters at various institutions are announced each week.—"J. M."

*MISCELLANEOUS.*

### Sterilisation of Gloves.

According to the latest accounts the following is the most economical and efficient method of sterilising rubber gloves without injuring them:—Wash the gloves in running water after using them, and dry them. Place them in a five-per-thousand solution of sulphuric acid, and leave them for ten or twelve hours, after which rinse them in salt solution, when they will be ready for use. Arnd and Rusca have been using this method for several years and are well pleased with it.

### Imitation Marble.

The polished slabs to which you refer are known as "Marosa," and are manufactured by Messrs. D. Anderson and Son, Limited, Roach Road Works, Old Ford, E. They are used in hospital theatres, bathrooms, etc., and make a wall covering that is easily kept clean; it is very durable, and low in cost. The slab is easily fixed to plaster, wood, or brick, and it may be cut or drilled without fear of splitting the material.—Lane.

### Maple Floors.

An answer to your inquiry respecting the treatment of maple floors can best be given by referring you to our issue of December 10, 1910, in which a report upon Hospital Construction by Mr. Webster, Superintendent of the Royal Victoria Hospital, Montreal, was quoted as follows:—"New floors treat with oil, then shellac, and afterwards two coats of varnish (Marnot). When thoroughly dry, wax with the following preparation:—Beeswax ½ lb., 1 oz. resin, 1 oz. pulv. alum, 2 pints turpentine, 1 tablespoonful Japan (dryer) turpentine. Mix the wax and resin over a fire, then remove from the heat. Add your turpentine and Japan, and your alum last. Then let it stand until it forms a paste. Apply the wax with waste and then brush."—N. M.

*ACKNOWLEDGMENTS.*

**C. E. J.**—We are glad to know that you appreciate the efforts of the Bureau on your behalf, and we shall be pleased to hear from you again should you require further assistance.

**B. R.**—We thank you for your suggestion, and for your kind offer to send us a letter for the Seaford Convalescent Home. The case has already been provided for, so that we do not now require the letter, but we are, nevertheless, much indebted to you for your prompt response to our appeal.

*Communications have been received and attended to from the following:—R. S. Campbell, J. A. Greene, L. A. Fisher, L. Whiteside, M. McIlraith, and E. Burrow.*

*The Editor is indebted for a copy of the report of St. Elizabeth's Home of Rest for Poor Ladies, Henfield.*

**We desire to inform numerous correspondents that we are unable to forward letters from or recommend any but our Approved Homes.**

### Editor's Notices.

**Contributions:**

Contributions should be written, or preferably typed, on one side of the paper only, and all articles sent in are accepted upon the distinct understanding that they are forwarded to THE HOSPITAL only.

The Editor cannot undertake to return MSS. not used, but when a stamped directed envelope is enclosed the MSS. may be returned if a special request is made.

Accepted articles and paragraphs of news will be paid for after publication at the scale rate.

**Address:**

To prevent delay all contributions and letters on editorial business must be addressed exclusively to the Editor, "The Hospital," 29 Southampton Street, Strand, London, W.C. It is important that this regulation shall be strictly observed.

**Correspondence:**

Correspondence on all subjects is invited. The name and address of correspondents must be given as a guarantee of good faith, but not necessarily for publication.

**Special Articles:**

Special articles are invited, and questions, inquiries, and paragraphs upon all matters relating to the work, administration and management of general, special, mental, fever, cottage and convalescent hospitals, sanatoria, homes, institutions, societies and organisations for the treatment or care of the sick, injured and dependants of all classes. Every matter affecting the interests and welfare of the staff of all grades working in these institutions will receive special consideration.

**Administrative Medicine, Research and Items of News:**

Special terms will be paid for approved articles on subjects in this wide field by experts who have made some section of it their special study and interest. We wish to give prominence to every movement tending to advance accuracy of diagnosis, efficiency in treatment, and the development of modern methods to eradicate disease and promote the welfare of the suffering.

**A Bureau of Information:**

We invite inquiries and applications for information and help in providing for that numerous class of sufferers who require special care or house-room which they cannot obtain in their own homes or secure for themselves. We cannot, however, prescribe, or recommend practitioners

**Books for Review:**

Publishers are particularly requested to send advance proofs of any new books of importance whenever possible, as the Editor has made arrangements to publish immediate reviews on a new plan.

**Photographs, Plans, Blocks, and Illustrations:**

It is requested that wherever possible MSS. may be accompanied by illustrations in any of the above forms. The name of the sender and of the article to which it belongs should be written on the back of each photograph, drawing, or block for purposes of identification.

**Local Papers:**

Newspapers containing reports or news paragraphs should be marked and addressed to the Sub-Editor.

**The Coupon System:**

A coupon will be found at the bottom of the third inside page of the cover each week. This coupon must be attached to every question or inquiry to which an answer is desired in THE HOSPITAL.

### To Help Our Readers.

THE ever-increasing circulation of THE HOSPITAL often renders it a matter of some difficulty to procure a copy of the current issue at the local newsagent's. By sending a subscription to the Journal, however, either through a bookseller or direct to the Publishing Offices you guard against the contingency of THE HOSPITAL being "sold out," and ensure a copy reaching you almost immediately upon publication.

When seeking a post this is a great help, since it enables you to send in your application without delay, an advantage which may secure you the appointment. In addition to this, as a subscriber, no matter how many times you may change your address, or in what part of the country you may be at the moment, a postcard will ensure that you receive THE HOSPITAL on the same day and at the same hour as when at home.

**Subscriptions** may begin at any time, and are payable in advance. Cheques and Post-Office Orders should be crossed London County and Westminster Bank, Covent Garden Branch, and made payable to the Manager of THE HOSPITAL.

RATES OF SUBSCRIPTION (payable in advance).

| | | |
|---|---|---|
| **UNITED KINGDOM.** | | |
| Three Months (including Postage) | ... ... ... ... | 2s. 0d. |
| Six Months do. | ... ... ... ... | 4s. 0d. |
| Twelve Months do. | ... ... ... ... | 6s. 6d. |
| **FOREIGN AND COLONIAL.** | | |
| Three Months (including Postage) | ... ... ... ... | 2s. 6d. |
| Six Months do. | ... ... ... ... | 5s. 0d. |
| Twelve Months do. | ... ... ... ... | 8s. 8d. |

*SUBSCRIPTION FORM.*

*Please send me* THE HOSPITAL *for*...............*months, commencing with your issue of*..........................., *for which please find enclosed*........................

*Name*............................................

*Address* ..........................................
..........................................

*Date*..................................

*To* ........................................ *Newsagent.*

### Manager's Notices.

**Letters relating to the publication, sale, and advertising departments of "The Hospital" must be addressed to The Manager, "The Hospital," The Hospital Building, 28 and 29 Southampto Street, London, W.C.**

**Advertisements:**

To ensure insertion, all advertisements for THE HOSPITAL must reach the Manager not later than Wednesday morning in each week. For scale of charges see pages v and 4.

**Remittances:**

**It is especially requested that remittances be made by Cheque or Postal Order, and in halfpenny stamps only when the amount is under sixpence.**

### HANDBOOKS

### For TRAINED WORKERS

POCKET-GUIDE SP SERIES

1/- net. 1/1 Post free.

**Bound in Limp Cloth.**
**Suitable Size for the Pocket.**

The works which comprise the S.P. Pocket Guide Series are intended for ready reference, consequently the aim of the Publishers has been to make them as concise and lucid as possible. Each book is written by an expert on the subject of which it treats, and the utmost reliance therefore can be placed in the information it imparts.

**Essentials of Fever Nursing.** By LYTTON MAITLAND, M.D. (Lond.), M.B., B.S., D.P.H. (Camb.).

**Manual and Atlas of Swedish Exercises.** *(With over 60 Illustrations.)* By THOMAS D. LUKE, M.D., F.R.C.S.

**Bandaging Made Easy.** *(Illustrated with over 90 Diagrams.)* By M. HOSKING, Sister-in-Charge, Tredegar House, Bow, E.

**How to Write and Read Prescriptions.** By LYTTON MAITLAND, M.D. (Lond.), M.B., B.S., D.P.H. (Camb.).

**Principal Drugs and their Uses.** By A PHARMACIST.

**Asepsis and How to Secure It.** By H. W. CARSON, F.R.C.S.

**Treatment after Operations.** *Rules for Nursing after General and Special Operations.* By MARY WILES.

**The Nurse's Duties before and during Operations.** By MARGARET FOX, Matron, Prince of Wales's General Hospital, Tottenham.

**Other works, in amplification of this Series, will be announced in due course.**

*Published by*

**The SCIENTIFIC PRESS, Ltd.**

**28/29 Southampton Street, STRAND, LONDON, W.C.**

## Figures and Tables

**Figure f1:**